# A Novel Approach to Preoperative Risk Stratification in Endometrial Cancer: The Added Value of Immunohistochemical Markers

**DOI:** 10.3389/fonc.2019.00265

**Published:** 2019-04-12

**Authors:** Vit Weinberger, Marketa Bednarikova, Jitka Hausnerova, Petra Ovesna, Petra Vinklerova, Lubos Minar, Michal Felsinger, Eva Jandakova, Marta Cihalova, Michal Zikan

**Affiliations:** ^1^Department of Gynecology and Obstetrics, University Hospital in Brno and Masaryk University, Brno, Czechia; ^2^Department of Internal Medicine – Hematology and Oncology, University Hospital in Brno and Masaryk University, Brno, Czechia; ^3^Department of Pathology, University Hospital in Brno and Masaryk University, Brno, Czechia; ^4^Faculty of Medicine, Institute of Biostatistics and Analyses, Masaryk University, Brno, Czechia; ^5^Institute of Biostatistics and Analyses, Faculty of Medicine, Masaryk University, Brno, Czechia

**Keywords:** endometrial cancer, ER, imaging method, L1CAM, PR, preoperative biopsy, p53, risk stratification

## Abstract

**Background:** The current model used to preoperatively stratify endometrial cancer (EC) patients into low- and high-risk groups is based on histotype, grade, and imaging method and is not optimal. Our study aims to prove whether a new model incorporating immunohistochemical markers, L1CAM, ER, PR, p53, obtained from preoperative biopsy could help refine stratification and thus the choice of adequate surgical extent and appropriate adjuvant treatment.

**Materials and Methods:** The following data were prospectively collected from patients operated for EC from January 2016 through August 2018: age, pre- and post-operative histology, grade, lymphovascular space invasion, L1CAM, ER, PR, p53, imaging parameters obtained from ultrasound, CT chest/abdomen, final FIGO stage, and current decision model (based on histology, grade, imaging method).

**Results:** In total, 132 patients were enrolled. The current model revealed 48% sensitivity and 89% specificity for high-risk group determination. In myometrial invasion >50%, lower levels of ER (*p* = 0.024), PR (0.048), and higher levels of L1CAM (*p* = 0.001) were observed; in cervical involvement a higher expression of L1CAM (*p* = 0.001), lower PR (*p* = 0.014); in tumors with positive LVSI, higher L1CAM (*p* = 0.014); in cases with positive LN, lower expression of ER/PR (*p* < 0.001), higher L1CAM (*p* = 0.002) and frequent mutation of p53 (*p* = 0.008).

Cut-offs for determination of high-risk tumors were established: ER <78% (*p* = 0.001), PR <88% (*p* = 0.008), and L1CAM ≥4% (*p* < 0.001). The positive predictive values (PPV) for ER, PR, and L1CAM were 87% (60.8–96.5%), 63% (52.1–72.8%), 83% (70.5–90.8%); the negative predictive values (NPV) for each marker were as follows: 59% (54.5–63.4%), 65% (55.6–74.0%), and 77% (67.3–84.2%). Mutation of p53 revealed PPV 94% (67.4–99.1%) and NPV 61% (56.1–66.3%). When immunohistochemical markers were included into the current diagnostic model, sensitivity improved (48.4 vs. 75.8%, *p* < 0.001). PPV was similar for both methods, while NPV (i.e., the probability of extremely low risk in negative test cases) was improved (66 vs. 78.9%, *p* < 0.001).

**Conclusion:** We proved superiority of new proposed model using immunohistochemical markers over standard clinical practice and that new proposed model increases accuracy of prognosis prediction. We propose wider implementation and validation of the proposed model.

## Introduction

Endometrial carcinoma (EC) is one of the most common female cancers. It predominantly has a favorable prognosis, due to the early onset of signs and symptoms such as postmenopausal bleeding or spotting, which lead to early-stage diagnosis in most patients and five-year overall survival rates of up to 85% (1). However, 20% of those EC patients who are estimated to be at low risk of recurrence will nevertheless recur while up to 50% of those designated “high-risk” will not (2, 3). It is clear the prognostic markers currently used (FIGO stage, tumor subtype, and histological grade) are far from optimal in terms of preoperative stratification of patients into low- or high-risk groups regarding surgical planning and adjuvant treatment.

One of the currently used prognostic markers is FIGO stage. This is obligatory and determined by transvaginal ultrasound of the pelvis (US). Computed tomography (CT) of the chest and abdomen is an imaging method of choice and is routinely used to exclude retroperitoneal lymphadenopathy and metastases in parenchymal organs. The other prognostic markers, histotype, and grade of tumor differentiation, are assessed from a biopsy obtained either by dilatation and curettage of the uterus or by hysteroscopy. Based on the established FIGO stage, histotype, and tumor grade, patients are divided into two groups regarding the recurrence risk. Low-risk patients are treated with surgery alone, consisting of hysterectomy and bilateral salpingo-oophorectomy, while high-risk patients undergo more aggressive surgical treatment, including pelvic (PLN) and/or paraaortic lymphadenectomy (PALN) with or without adjuvant radiotherapy or chemotherapy. An aggressive therapeutic approach is associated with significantly higher side effects, such as increased blood loss, risk of thrombosis, infection, lymphoceles, lymphatic ascites, and lymphedema (4, 5).

The discovery of new histotype-specific and prognostic biomarkers for better stratification into high- or low-risk EC seems to be urgently needed in order to avoid over- or undertreatment of EC patients. The results of studies on potential new biomarkers assessed immunohistochemically (IHC), related to EC patient prognosis, were recently published. L1 cell adhesion molecule (L1CAM) overexpression and the loss of estrogen receptors (ER) and/or progesterone receptors (PR) are associated with poor prognosis and a high risk of relapse and death (6–9). Mutations of the tumor protein p53 are associated with L1CAM expression, but not universally (10). However, to our best knowledge, neither the significance of L1CAM, ER, and PR expression nor knowledge of their relevant cut-offs together with determination of p53 mutation status in preoperative biopsies for pretreatment stratification into low- or high-risk have been established yet. No IHC biomarkers from preoperative biopsy are currently routinely used in the decision-making process for EC management.

The purpose of this study was to evaluate the clinical usefulness and added value of preoperatively assessed IHC biomarkers L1CAM, ER, PR, and p53 in differentiation between low- and high-risk EC patients through comparison of the current clinical practice model with a proposed model that includes immunohistochemical markers. The secondary objective of our study was to evaluate the correlation of IHC biomarkers with specific clinical (according to preoperative ultrasound and CT chest/abdomen) and pathological parameters.

## Patients and Methods

### Patients

Patients undergoing surgical treatment for histologically proven or suspicious EC in the oncogynecological center of University Hospital Brno, Czech Republic, from January 2016 to August 2018 were consecutively included. The study was approved by the Institutional Ethical Board as was a version of written informed consent regarding tissue and clinical data use for scientific purposes obtained from each eligible patient.

### Preoperative Imaging

All patients underwent a clinical examination, preoperative ultrasound staging examination, and CT of the chest/abdomen according to the local guidelines (11, 12). Each patient underwent both a transabdominal and a transvaginal US scan within 14 days before a board discussion led by one of the two oncogynecologists experienced in the field of US diagnostics in gynecologic oncology. Each US examination was immediately described in a written report; these reports were used for study analysis. Descriptions and examination reports were based on the standards applied by our center (13). During US staging examination of the uterine cavity, myometrium and cervix and pelvic lymph nodes were carefully assessed in every patient to describe the local extent of the tumor (14, 15).

Each patient underwent a CT scan of the chest, abdomen, and pelvis within 14 days before board discussion and admission to the operating theater. CT was performed with oral and intravenous contrast in order to exclude bowel wall implants, parenchymatous metastasis, and pathological lymphadenopathy. When lymph nodes measured >1 cm in the shorter axis or morphological changes as a rounded shape or necrosis were observed, tumor involvement was marked as suspicious.

### Risk Stratification and Clinical Management

The extent of the surgery was determined by the multidisciplinary board after dividing patients into the low- or high-risk group based on clinical staging and the preoperative histopathological examination and determination of the histotype and grading. The low-risk group was defined as endometrioid or mucinous carcinoma TNM stage cT1a or cT1b, grade 1 and/or endometrioid or mucinous carcinoma TNM stage cT1a, grade 2, all without clinical or imaging evidence of lymphadenopathy (cN0) or distant metastases (cM0). Patients were defined as high-risk unless these low-risk criteria were met. Type A radical hysterectomy with bilateral salpingo-ophorectomy was performed in all patients (16). Systematic pelvic and paraaortic lymphadenectomy was performed in the high-risk group only; in high-grade serous uterine cancer cases, total omentectomy and appendectomy were added to the staging procedure. The definitive histopathological examination was provided by one of three pathologists with experience in gynecological malignancies and contained data about stage, histotype, and grade, lymphovascular space involvement (LVSI), and measures of the IHC expression of markers L1CAM, ER, PR, and p53. Based on final histopathological findings, the patients were once again stratified into low- or high-risk groups based on the same preoperative criteria (i.e., irrespective of known IHC status of ER, PR, L1CAM, and p53) and, thereafter, decisions regarding adjuvant treatment and follow-up were made by the multidisciplinary board.

### Clinical Data

Age, results of US and CT scan with respect to depth of myometrial invasion, cervical involvement, lymphadenopathy, parenchymal organ involvement, and pathological data from biopsies (histotype, grading, IHC status of L1CAM, ER, PR, p53) were recorded.

### Tissue and Immunohistochemistry Analysis

**Figure 1:**
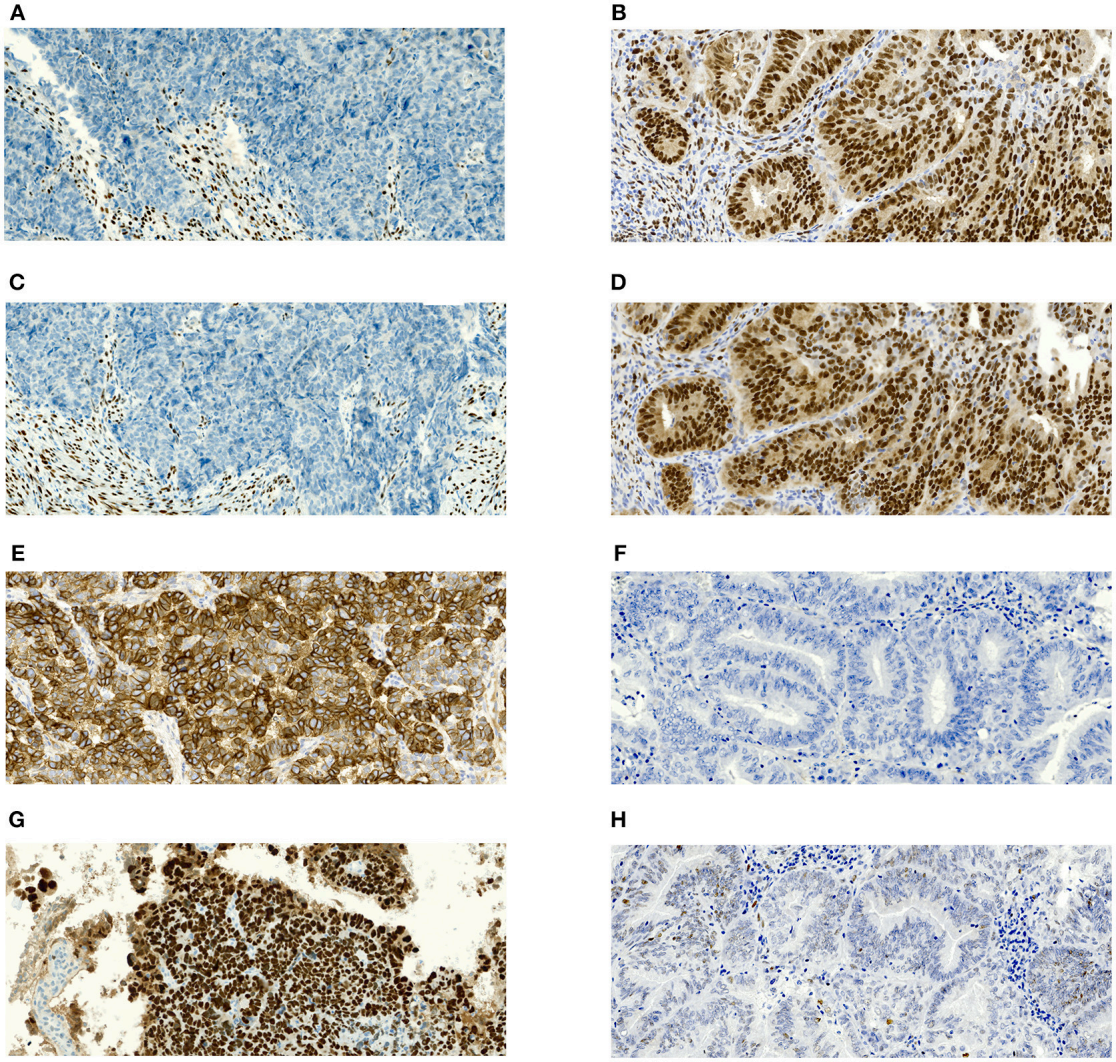
Microphotographs showing representative examples of immunohistochemical expression of estrogen receptors (ER), progesterone receptor (PR), L1CAM and p53 in tissue specimens of endometrial carcinomas. Magnification 100x. **(A)** Complete negativity of ER expression in serous carcinoma with 0% cells positive; **(B)** Complete negativity of PR expression in serous carcinoma with 0% cells positive; **(C)** Strong diffuse membranous positivity of L1CAM expression in serous carcinoma with 100% cells positive; **(D)** p53 nuclear overexpression (mutant pattern) in serous carcinoma; **(E)** Nuclear positivity of ER expression in grade 1 endometrioid carcinoma with almost 100% cells positive; **(F)** nuclear positivity of PR expression in grade 1 endometrioid carcinoma with almost 100% cells positive; **(G)** complete negativity of L1CAM expression in grade 1 endometrioid carcinoma with 0% cells positive; **(H)** p53 wildtype immunohistochemical pattern in grade 1 endometrioid carcinoma.

All hematoxylin and eosin-stained slides were read by one of three experienced gynecological histopathologist to confirm histological subtype, grade, and (definitive excision specimen) stage and the presence or absence of LVSI. The evaluator was blinded to patient characteristics. All specimens were assessed according to the WHO Classification of Tumors of Female Reproductive Organs, 2014 (17). No additional later review of the slides was performed for the purpose of this study because it would not copy our real clinical practice. Immunohistochemical staining was performed on formalin-fixed and paraffin-embedded (FFPE) tissue sections. Immunohistochemistry for ER (clone SP1, product no. RBK 018-05, Zytomed, dilution 1:300), PR (clone 16, product no. NCL-L-PGR-312, Novocastra, dilution 1:80), L1CAM/CD171 (clone 14.10, product no. 826701, BioLegend, dilution 1:100), and p53 (clone DO-7, product no. M7001, DAKO, dilution 1:300) were performed using an automatic immunostainer (BenchMark Ultra, Ventana Medical Systems, Tucson, AZ, USA) according to the manufacturer's instructions. For ER, PR, and p53, only nuclear staining was scored as positive. Positivity of L1CAM was defined as distinct membrane staining. For ER, PR, and L1CAM, the percentage of positive tumor cells was assessed. p53 was classified into wild type or mutant (excessive = strong diffuse overexpression in more than 90% of tumor cells or completely negative) phenotypes. Representative microphotographs of the expression of estrogen receptor (ER), progesterone receptor (PR), L1CAM and p53 in serous (high risk) and grade 1 endometrioid (low risk) carcinoma are shown in Figures 1A–H.

### Statistical Analysis

Categorical data were summarized using absolute and relative frequencies and compared by Fisher's exact test. Continuous variables were summarized as median with 10 and 90^th^ percentile and tested by the Mann-Whitney *U*-test.

A model for the best classification of final risk was built using the CHAID growing method with crossover validation. Misclassification cost for wrongly determining high-risk patients as low-risk was set twice higher because of the preference for the correct high-risk group EC patient determination.

The success of risk-group classification was evaluated using four standard measures: (i) *sensitivity* is the ability of the test to correctly identify those with an occurrence of the assessed marker (true positive rate), whereas (ii) *specificity* is the ability of the test to correctly identify those without an occurrence of the assessed marker (true negative rate), (iii) *positive predictive value* (PPV) is the probability that the marker is present when the test is positive, whereas (iv) *negative predictive value* (NPV) is the probability that the marker is not present when the test is negative. All these statistics were accompanied by 95% confidence intervals (CI).

The comparison of sensitivities and specificities of the two binary diagnostic tests in a paired study design was performed using McNemar's test with continuity correction. Differences in (positive and negative) predictive values of two binary diagnostic tests were tested using a generalized score statistic proposed by Leisenring, Alonzo, and Pepe (18). All tests were performed as two-sided at the significance level 0.05. Analyses were done in IBM SPSS Statistics and R.

## Results

### Clinical and Histopathological Characteristics

From January 2016 to August 2018, 132 patients underwent surgical treatment for EC in the oncogynecological center of University Hospital Brno, Czech Republic, and have been consecutively enrolled in the study. The median age was 66 years. According to ultrasound and CT staging, before operation 95 patients (72%) were evaluated as FIGO stage IA, while 25 (19%) were stage IB, 5 (4%) stage II, 3 (2%) stage III, and 4 (3%) were at an unknown stage. Preoperative biopsy was available for all 132 patients; 102 patients had endometrioid cancer, of whom 50 (49%) had endometrioid or mucinous carcinoma grade 1 (EG1), 45 (44%) grade 2 (EG2), 6 (6%) grade 3 (EG3), and one had a non-diagnostic grade. Seventeen (13%) patients were diagnosed with non-endometrioid carcinoma (NEC). Furthermore, there were eight cases (6%) of endometrial intraepithelial neoplasia (EIN) in preoperative biopsy (Table 1). Based on both histological and clinical findings, 94 (71%) patients were preoperatively classified as low-risk and 38 (29%) as high-risk by current model and, consequently, the recommendation for the extent of surgery was issued. In the high-risk group, PLN and PALN were performed for 26 (20%) patients, apart from hysterectomy and bilateral salpingo-oophorectomy. After the surgical procedure and definitive histopathological examination, 82 (62%) patients were at FIGO stage IA, 17 (13%) at FIGO stage IB, 19 (14%) FIGO stage II, 12 (9%) FIGO stage III and two (2%) with FIGO stage IV. Regarding endometrioid or mucinous carcinoma grade, 30 (28%) were at EG1, 69 (65 %) at EG2, 8 (7 %) EG3, 20 (15%) NEC, and 2 (2%) EIN. In contrast to the preoperative risk determination, the final post-operative stratification in risk groups was as follows: 70 (53%) low-risk patients and 62 (47%) high-risk patients (Table 1).

**Table 1:** Patients' clinical and histopathological characteristics.

**Age at diagnosis**	**66 (50–78)**		***p*-value**
**Histology**	**Preoperative (biopsy or imaging)**	**Final specimen**	
Endometrioid (incl. mucinous)	102 (77%)	107 (81%)	0.216
Non-endometrioid	17 (13%)	20 (15%)	
Serous	6 (35%)	2 (10%)	
Clear cell	4 (24%)	2 (10%)	
Carcinosarcoma	1 (6%)	2 (10%)	
Undifferentiated carcinoma	2 (11%)	3 (15%)	
Mixed carcinoma	4 (24%)	11 (55%)	
EIN	8 (6%)	2 (2%)	
Non-diagnostic	5 (4%)	3 (2%)	
**GRADE (ONLY ENDOMETRIOID)**			
G1	50 (49%)	30 (28%)	0.006
G2	45 (44%)	69 (65%)	
G3	6 (6%)	8 (7%)	
Non-diagnostic	1 (1%)		
**MYOMETRIAL INVASION**			
<50%	96 (73%)	93 (70%)	0.557
≥50%	33 (25%)	39 (30%)	
Unknown	3 (2%)		
**CERVICAL INVASION**			
Yes	9 (7%)	24 (18%)	0.015
No	120 (91%)	108 (82%)	
Unknown	3 (2%)		
**LYMPHADENOPATHY**			
Yes	3 (2%)	9 (7%)	0.070
No	129 (98%)	123 (93%)	
**TUMOR BOARD DECISION**			
Low-risk EC	94 (71%)	70 (53%)	<0.001
High-risk EC	38 (29%)	62 (47%)	

*values denote median (10-90th percentile) or n (%); p-values of chi-square or McNemar's test; G, grade; EC, endometrial cancer*.

### Immunohistochemical Characteristics

The expression of markers ER, PR, L1CAM, and p53 status were immunohistochemically evaluated from the specimen obtained both by diagnostic procedure and definitive surgery. The correlation of IHC markers between preoperative examination and definitive histopathological findings was statistically significant for all of the evaluated markers (*p* < 0.005). In the preoperative specimen, the expression was evaluable in 98 patients for ER and PR, 97 for L1CAM, and 98 for p53 mutational status; results are listed in Table 2.

**Table 2:** Immunohistochemical biomarkers in preoperative biopsies.

**IHC markers**	**Overall values (*n* = 98)**	**EG1 (*n* = 36)**	**EG2 (*n* = 37)**	**EG3 (*n* = 9)**	**EIN (*n* = 3)**	**NEC (*n* = 13)**
ER (%), *n* = 98	86 (27)	95 (15)	96 (8)	71 (34)	100 (0)	44 (42)
	99 (40–100)	100 (90–100)	99 (85–100)	80 (0–100)	100 (100–100)	30 (0–100)
PR (%), *n* = 98	73 (34)	88 (21)	79 (28)	48 (39)	87 (23)	28 (33)
	90 (5–100)	99 (60–100)	95 (30–100)	70 (0–95)	100 (60–100)	20 (0–85)
L1CAM (%), *n* = 97	16 (29)	2 (4)	7 (13)	33 (35)	0 (1)	72 (33)
	3 (0–70)	1 (0–8)	3 (0–15)	30 (0–100)	0 (0–1)	85 (15–100)
p53, *n* = 98						
Mut	16 (16.3%)	1 (2.8%)	2 (5.4%)	2 (22.2%)	0 (0%)	11 (84.6%)
Wt	75 (76.5%)	35 (97.2%)	32 (86.5%)	4 (44.4%)	2 (66.7%)	2 (15.4%)
Non-specific	7 (7.1%)	0 (0%)	3 (8.1%)	3 (33.3%)	1 (33.3%)	0 (0%)

*Values denote mean (SD) and median (10–90th percentile) or n (%); means and SD are shown only for exploratory purpose since data are not normally distributed; IHC, immunohistochemical markers; ER, estrogen receptors; PR, progesterone receptors; L1CAM, L1cell adhesion molecule; mut, mutated; wt, wild type; EG1, endometrioid or mucinous cancer, grade 1; EG2, endometrioid or mucinous cancer, grade 2; EG3, endometrioid or mucinous cancer, grade 3; EIN, endometrioid intraepithelial neoplasia; NEC, non-endometrioid cancer*.

### Correlation of IHC Markers With Disease Extent (FIGO Staging)

The correlation was assessed between IHC markers in preoperative tissue samples and the final histopathological findings (e.g., myometrial invasion, cervical, and lymph node involvement). Moreover, the correlation with LVSI was evaluated because LVSI is one of the important markers for adjuvant treatment strategy decisions. There were statistically significant lower levels of ER (*p* = 0.024) and PR (0.048) and higher levels of L1CAM (*p* = 0.001) in tumors with myometrial invasion >50%. In tumors with cervical involvement, a significantly higher expression of L1CAM was observed (*p* = 0.001), while differences among levels of ER (*p* = 0.236) and PR (*p* = 0.108) did not reach statistical significance. PR were significantly lower (*p* = 0.014) and L1CAM higher (*p* = 0.014) in tumors with positive LVSI. In patients with positive LN, levels of ER and PR were lower (*p* = 0.001 and *p* < 0.001, respectively); on the other hand, levels of L1CAM were higher (*p* = 0.002) and the mutation of p53 more frequent (*p* = 0.008), see Table 3.

**Table 3:** Correlation of IHC markers from preoperative biopsy with staging after surgery.

	**ER (%)** **(*N* = 98)**	**PR (%)** **(*N* = 98)**	**L1CAM (%)** **(*N* = 97)**	**p53 mut** **(*N* = 16)**	**P53 wt** **(*N* = 75)**
Myometrial invasion	*p* = 0.024	*p* = 0.048	*p* = 0.001	*p* = 0.382	
<50% (*N* = 66)	88 (26) 100 (60–100)	77 (31) 95 (20–100)	14 (29) 1 (0–70)	9 (14.8%)	52 (85.2%)
≥50% (*N* = 32)	83 (30) 98 (40–100)	65 (38) 82.5 (0–100)	20 (29) 5 (1–70)	7 (23.3%)	23 (76.7%)
Cervical involvement	*p* = 0.236	*p* = 0.108	*p* = 0.001	*p* = 0.070	
Yes (*N* = 19)	82 (31) 95 (0–100)	60 (39) 70 (0–100)	32 (38) 8 (1–95)	6 (35.3%)	11 (64.7%)
No (*N* = 79)	88 (26) 99 (40–100)	76 (32) 90 (15–100)	12 (26) 2 (0–50)	10 (13.5%)	64 (86.5%)
LN (lymph node) metastases	*p* = 0.001	*p* < 0.001	*p* = 0.002	*p* = 0.008	
Yes (*N* = 6)	43 (47) 35 (0–95)	11 (20) 0.5 (0–50)	60 (36) 70 (4–100)	4 (66.7%)	2 (33.3%)
No (*N* = 92)	89 (23) 99 (70–100)	77 (31) 92.5 (20–100)	13 (27) 2 (0–50)	12 (14.1%)	73 (85.9%)
LVSI	*p* = 0.111	*p* = 0.014	*p* = 0.014	*p* = 0.260	
Yes (*N* = 14)	74 (41) 95 (0–100)	48 (43) 45 (0–100)	23 (32) 6 (2–70)	4 (22.2%)	10 (55.6%)
No (*N* = 84)	89 (24) 99 (60–100)	77 (31) 93 (20–100)	15 (29) 2 (0–70)	12 (13.0%)	65 (70.7%)

*Values denote mean (SD) and median (10-90th percentile) or n (%), p-value of Mann-Whitney U-test or Fisher's exact test; means and SD are shown only for exploratory purpose since data are not normally distributed; LVSI, lymphovascular space involvemen; mut, mutated; wt, wild type*.

### The Precision of EC Risk Stratification Based on Markers Currently Used in Clinical Praxis

Concerning depth of myometrial invasion, we classified all the patients in whom invasion reached ≥50% as high-risk (*n* = 36) and the patients with invasion <50% as low-risk (*n* = 96). Our approach to preoperative risk stratification of EC patients revealed a sensitivity of 48% and specificity of 89% in terms of high-risk group determination. Taking all current standard prognostic markers together, it can be concluded that, whereas low-risk EC patients are preoperatively classified with relatively high accuracy (62/70, 82%), the determination of high risk is far from optimal, since more than half of EC patients with actual high-risk disease were established as low-risk. See Table 4.

**Table 4:** Accuracy of low-/high-risk group classification according to current practice.

	**Final risk**				
	**Low-risk**	**High-risk**	**Total *N***	**Sensitivity (95% CI)** **Specificity (95% CI)**	**PPV (95% CI)** **NPV (95% CI)**
Current model: low-risk	62	32	94	48.4% (35.5–61.4%)	78.9% (65.0–88.3%)
Current model: high-risk	8	**30**	38	88.6% (78.7–94.9%)	66.0% (60.0–71.4%)
Total *N*	70	62	132		

*CI, confidence interval; PPV, positive predictive value; NPV, negative predictive value. Bold values are significant*.

### The Accuracy of EC Risk Stratification by Using IHC Markers

**Figure 2:**
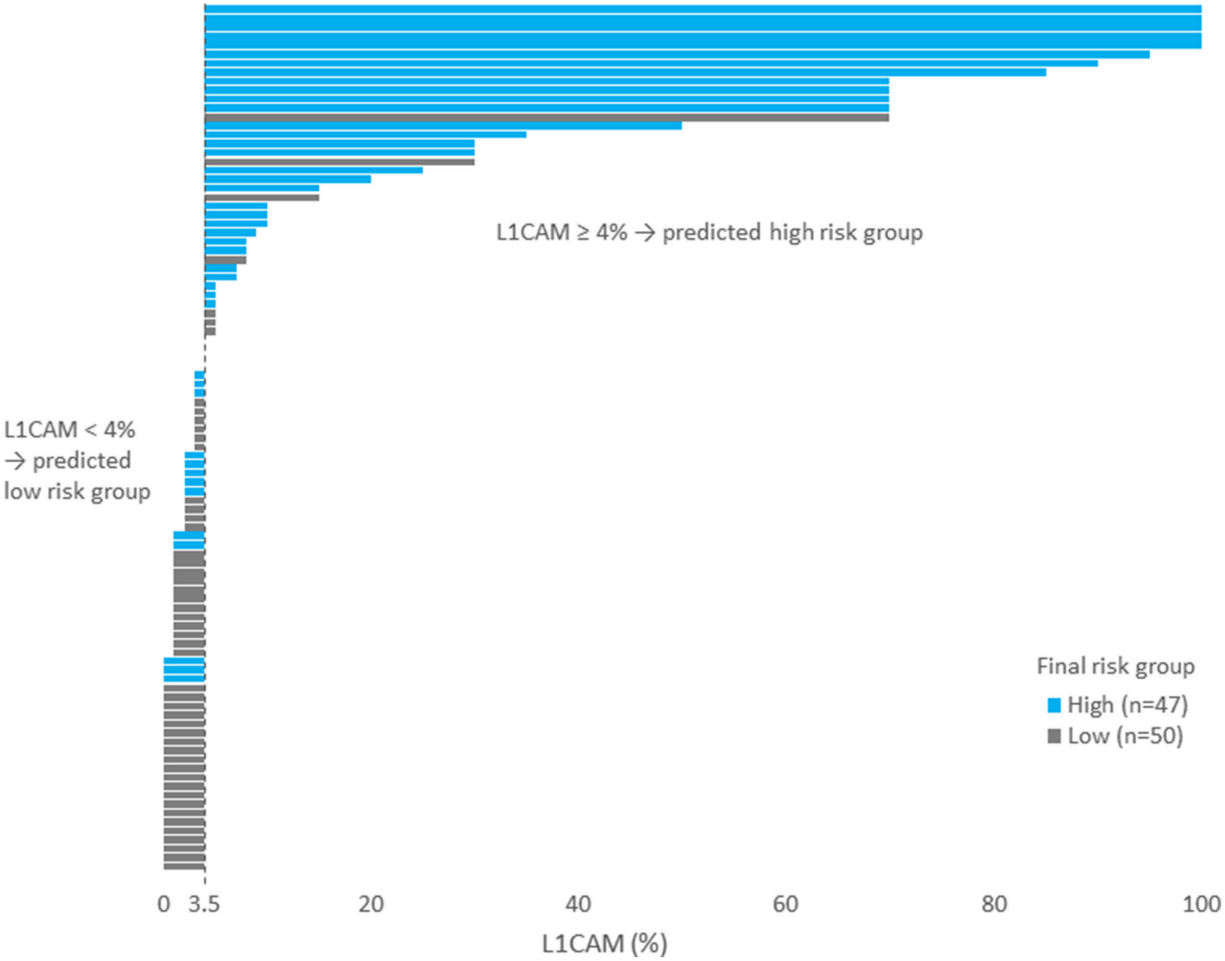
Each patient is represented by one bar. Patients on the left part of the figure (i.e., with L1CAM<4) was predicted as low risk. Blue bars represent wrongly predicted patients here. On the contrary, gray bars on the right side of the figure represent patients wrongly predicted as high risk.

IHC markers were assessed in a preoperative tumor sample, and optimal cut-offs for continuous markers (obtained preoperatively) were designed using ROC analyses. At a cut-off for ER <78% (*p* = 0.001), PR <88% (*p* = 0.008), and L1CAM ≥4% (*p* < 0.001), high-risk tumors were determined with a sensitivity of 28% for ER (15.6–42.6%), 62% for PR (46.4–75.5%), and 72% for L1CAM (57.4–84.4%). Specificity was 96% (86.5–99.5%), 68% (52.1–79.2%), and 86% (73.3– 94.2%), respectively. The PPV was 87% for ER (60.8–96.5%), 63% for PR (52.1–72.8%), and 83% for L1CAM (70.5–90.8%); the NPV for each marker were as follows: 59% (54.5–63.4%), 65% (55.6–74.0%), and 77% (67.3–84.2%), respectively. The sensitivity of p53 mutated status (*p* < 0.001) for high-risk detection was low (34%), but the specificity was high (98.0%, CI 88.7–99.9%), which represents PPV 94% (67.4–99.1%) and NPV 61% (56.1–66.3%), respectively. If p53 mutated, there was a high probability the patient fit into the high-risk group (15 out of 16 patients in our series). As far as accuracy of high-risk determination in the largest number of patients was concerned, the marker L1CAM seemed to be the most robust: 34 from 41 patients who had L1CAM values ≥4% were classified as high-risk (Table 5, Figure 2).

**Table 5:** Correlation of IHC with final EC risk stratification.

	**Final risk**					
	**Low-risk**	**High-risk**	**Total *N***	***p*-value**	**Sensitivity (95% CI)** **Specificity (95% CI)**	**PPV (95% CI)** **NPV (95% CI)**
ER < 78 (high-risk)	2	**13**	15	0.001	27.7% (15.6–42.6%)	86.7% (60.8–96.5%)
ER 78+ (low-risk)	49	34	83		96.1% (86.5–99.5%)	59.0% (54.5–63.4%)
Total *N*	51	47	98			
PR < 88 (high-risk)	17	**29**	46	0.008	61.7% (46.4–75.5%)	63.0% (52.1–72.8%)
PR 88+ (low-risk)	34	18	52		66.7% (52.1–79.2%)	65.4% (55.6–74.0%)
Total *N*	51	47	98			
L1CAM < 4 (low-risk)	43	13	56	<0.001	72.3% (57.4–84.4%)	82.9% (70.5–90.8%)
L1CAM 4+ (high-risk)	7	**34**	41		86.0% (73.3–94.2%)	76.8% (67.3–84.2%)
Total *N*	50	47	97			
p53 mut (high-risk)	1	**15**	16	<0.001	34.1% (20.5–49.9%)	93.8% (67.4–99.1%)
P53 wt (low-risk)	46	29	75		97.9% (88.7–99.9%)	61.3% (56.1–66.3%)
Total *N*	47	44	91			

*p-value of Fisher's exact test; CI, confidence interval; PPV, positive predictive value; NPV, negative predictive value; mut, mutated; wt, wild type. Bold values are significant*.

### The Added Value of IHC Markers for Improvement of EC Risk Stratification

**Figure 3:**
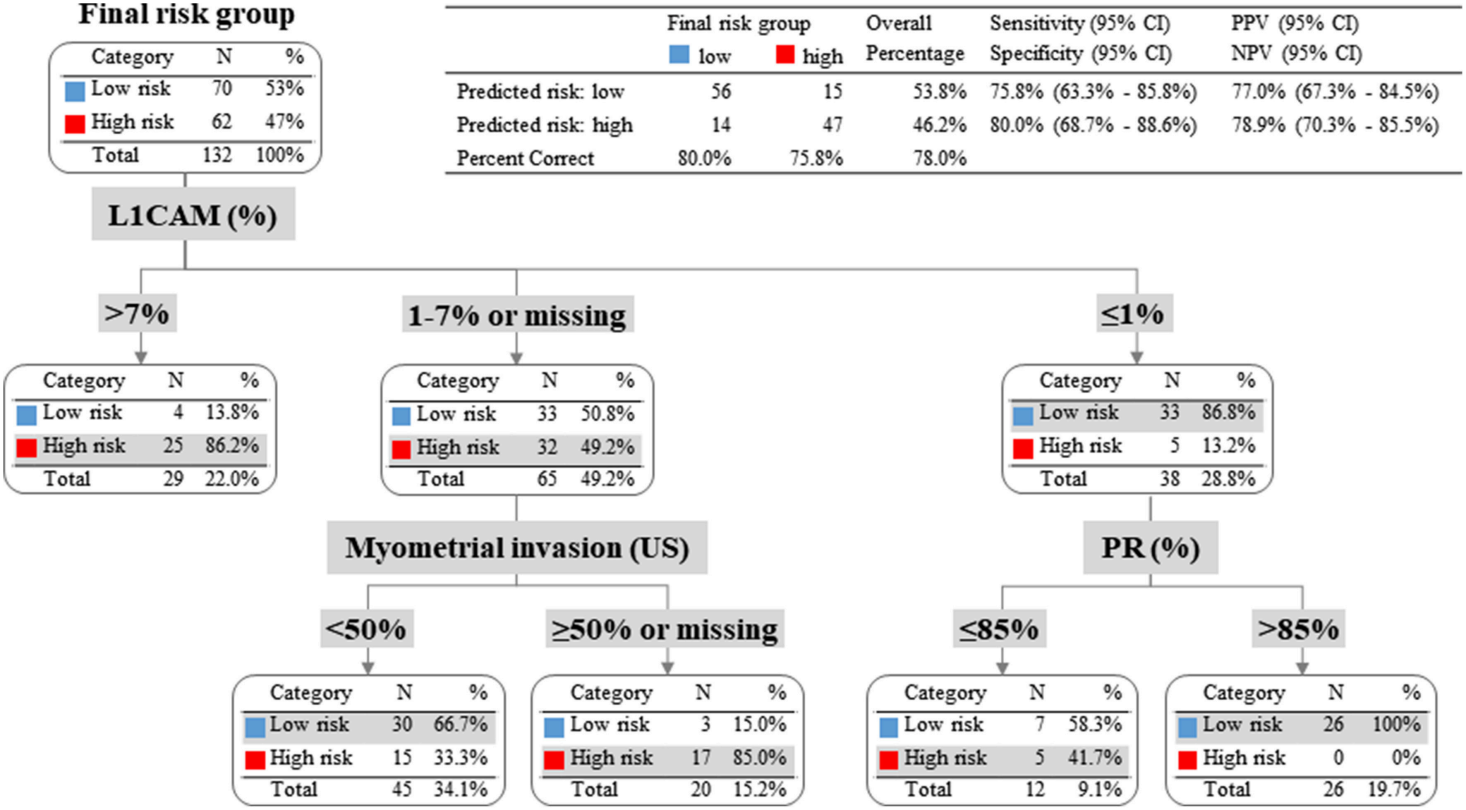
Patients with L1CAM positivity >4% were identified as high risk; in fact 86% of them were high-risk indeed. Patients with L1CAM value from 1 to 4% were further divided according to myometrial invasion. In case of myometrial invasion <50% they were stratified as low-risk, on the contrary in case of myometrial invasion >50% or unknown they were classified as high-risk. Patients with L1CAM <1% were further disaggregated by PR value. In case of PR >85% they were identified as low-risk (with success rate of 100%), on the other hand in case of PR ≤85% they were stratified as high-risk according to clinical preference for more precise high-risk group determination even with the expectation of higher false positivity.

To evaluate whether IHC markers would contribute to more accurate stratification of EC patients into risk groups, a model consisting of parameters obtained by imaging methods (myometrial invasion, cervical involvement, lymph node involvement), histology (endometrioid or mucinous vs. non-endometrioid), grade and IHC markers (ER, PR, L1CAM, p53) was introduced. According to risk stratification, the following parameters have been shown as statistically significant and crucial for the model: L1CAM, PR, and myometrial invasion. The procedure of classification is shown in Figure 3. The overall EC risk stratification success was 78% for this model. Successful group inclusion was observed for 80% of the low-risk patients (56/70), and for 76% of high-risk patients (47/62 patients).

### New Model in Comparison to Current Practice

**Figure 4:**
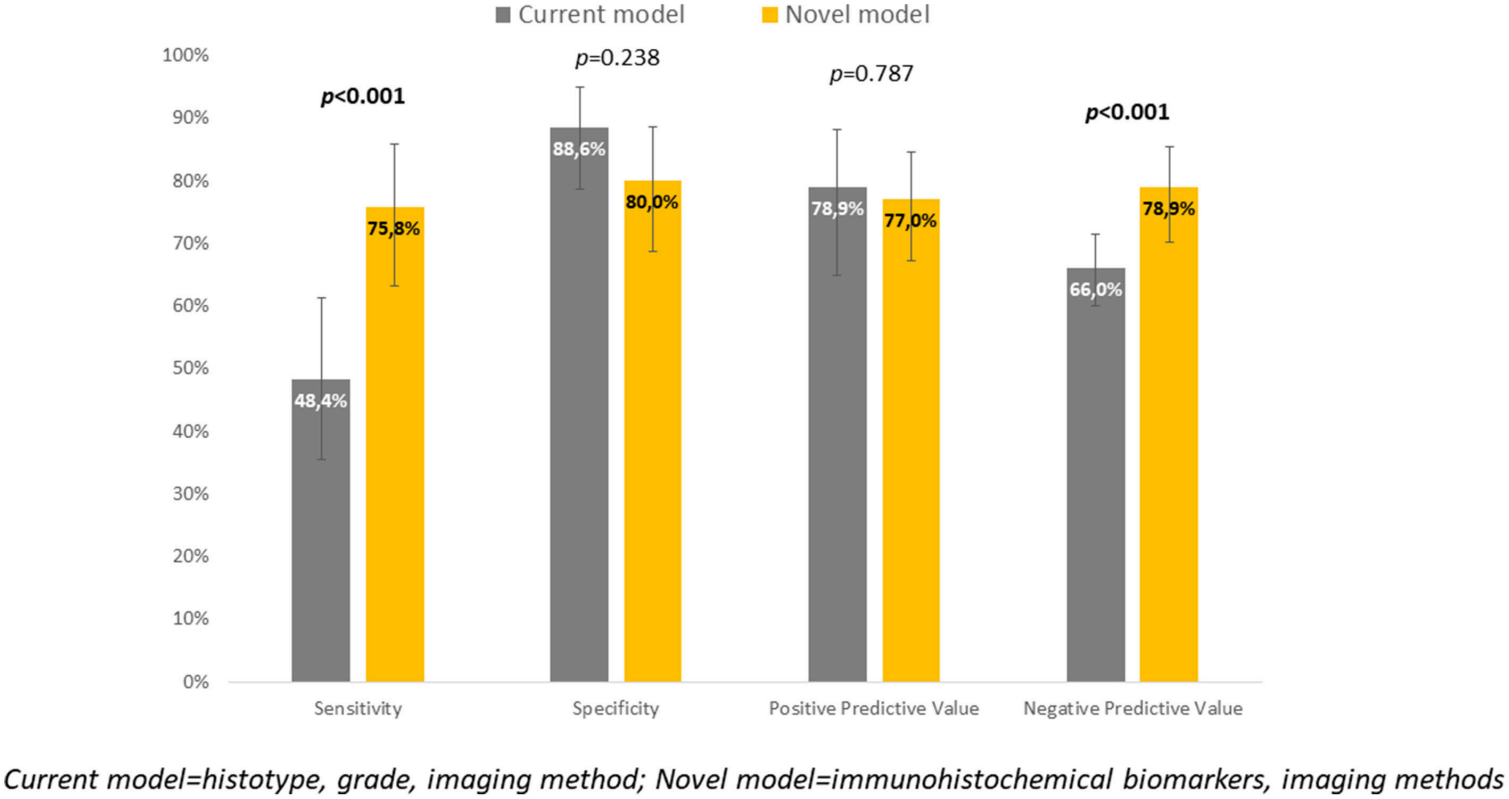
Comparison of current and novel model for prediction of risk tumor type.

Immunohistochemical markers included in the current diagnostic practice would significantly improve sensitivity (48.4 vs. 75.8%, *p* < 0.001) associated with a slightly, statistically non-significant decrease in specificity (to 80%, *p* = 0.238). Positive predictive values were similar for both methods, while negative predictive value (i.e., the probability of extremely low risk in negative test cases) was significantly improved (66 vs. 78.9%, *p* < 0.001) (Table 6, Figure 4).

**Table 6:** Comparison of standard and new diagnostic approaches.

		**Final risk: Low**			**Final risk: High**		
		**Current model: low-risk**	**Current model: high-risk**	**Total**	**Current model: low risk**	**Current model: high risk**	**Total**
Novel model (IMG+IHC)	Low	50 (71.4%)	**6 (8.6%)**	56 (80%)	15 (24.2%)	**0 (0%)**	15 (24,2%)
	High	**12 (17.1%)**	2 (2.9%)	14 (20%)	**17 (27.4%)**	30 (48.4%)	47 (75,8%)
Total		62 (88.6%)	8 (11.4%)	70 (100%)	32 (51.6%)	30 (48.4%)	62 (100%)

*IMG, imaging method; IHC, immunohistochemical markers; current model, histology, grading, imaging method. Bold values are significant*.

## Discussion

The determination of an appropriate surgery and its adequate extent is a crucial part of treatment in newly diagnosed EC patients and significantly differs between the high- and low-risk groups. Existing models determining patient risk are based on the synthesis of information obtained from the result of preoperative biopsy (histotype, grading) and imaging methods. Based on the results, patients are included in a risk group before the surgery and so only hysterectomy and salpingo-oophorectomy are indicated, or the procedure is extended by pelvic and para-aortic lymphadenectomy. Proper preoperative inclusion of a patient in the risk group is crucial for her treatment and overall survival and is a clinically crucial question.

Other approaches to assess the biological behavior of endometrial cancers are under development. Several research groups have defined immunohistochemical and/or mutation profiles to allow distinguishing endometrial cancer subtypes. The Cancer Genome Atlas (TCGA) project provided the most comprehensive molecular study on endometrial cancer so far. They identified four group with distinct molecular changes that correlate with progression free survival – *POLE* (Polymerase Epsilon subunit) ultramutated, MSI (microsatellite instability) hypermutated, copy-number low, and copy-number high (19). This approach allows objective categorization of endometrial cancers, however, methodologically remains costly, complex and unsuitable for wider clinical application.

Others introduced a concept of sentinel lymph node detection in endometrial cancer patients. The large prospective study led by Rossi (20) showed very high sensitivity (97.2%) and low false negativity rate (3%) for sentinel lymph node (SLN) detection. SLN detection concept is based on low risk of paraaortic lymph nodes involvement in patients with negative pelvic lymph nodes (21). However, controversy regarding sentinel lymph node detection in high-risk disease and management of low volume nodal disease on ultrastaging still remains.

In many centers, frozen section of uterus is still standard-of-care in terms to confirm or to more specify type and grade of the tumor. Accuracy of frozen section histopathological evaluation is, however, comparable to imaging methods and interobserver agreement regarding both the categories, type and grade, is poor (22–26).

The current development of risk prediction model is mainly focused on combination of imaging and molecular predictors, as our study does. In 2014, Van Holsbeke et al. published a study that externally validated two mathematical models of preoperative risk group prediction in a particular patient (27). The models were based on histology, grading, and the preoperative sonographic evaluation of tumor invasion into the myometrium and cervix. Both models achieved sensitivity of 78–83% and specificity of 68–72% in the detection of high-risk EC patients. In our study, the current clinical model reliably determined low-risk patients (correctly in 83% of cases), while only 8 (11%) patients in the study were false positives included in the high-risk group. The current model preoperatively stratified patients to high-risk with sensitivity of only 48% (35.5–61.4%) and specificity 89% (78.7–94.9%), NPV 66% (60.0–71.4%), and PPV 79% (65.0–88.3%) (Table 4).

A number of ultrasound studies have been published for the assessment of individual staging parameters to determine the depth of tumor invasion into the myometrium, with ultrasound sensitivity from 61 to 93% and specificity from 71 to 92%, when performed by an expert sonography specialist (28–32). The sensitivity reported in the evaluation of tumor invasion in cervical stroma was lower, from 25 to 93%, and specificity from 85 to 99% (30, 33, 34). In a study utilizing expert sonography in a specialized center, Fruhauf et al. reported a PPV of 67.6% and NPV of 83.3% for the detection of deep myometrial invasion and PPV of 60.0% and NPV of 88.1% in the detection of tumor affection of the uterine cervix. According to a recent meta-analysis of 18 studies, CT sensitivity is 47% and specificity 93%; as for ultrasound, sensitivity is 55% and specificity up to 85% for the detection of malignant lymphadenopathy (35). In our group of 132 female patients, the invasion of the tumor to half the thickness of the myometrium was determined correctly in 90%; a false-negative result in the high-risk group of patients was reported in 39 cases. CT did not detect pathological lymphadenopathy in six cases out of nine. A total of nine patients were classified as false positives on the basis of US and CT as validated by the definitive histology.

Studies showing the discrepancy between histology obtained from preoperative curettage or hysteroscopy and definitive histological findings have been published (36–38). On the other hand, there are studies showing good concordance between histology, grade, and immunohistochemical staining in curettage and hysterectomy samples (39, 40). We confirmed that the preoperatively determined histological type, grade, and immunohistochemical biomarkers L1CAM, ER, PR, p53 correlated with the final preparation.

In our study, 70 (70/132) patients were classified as low-risk and 62 (62/132) as high-risk. According to the pre-operative staging, PLN and PALN were performed in 26 patients (20%) in our cohort. However, if the definitive risk were known, staging lymphadenectomy would be performed in all 62 patients in the high-risk group (47%). Due to an inappropriate staging surgery, patients underwent repeated surgery or adjuvant radiotherapy, which may have been avoided if a complete surgical staging with negative histological findings of the presence of the tumor in the lymph nodes had been performed. On the contrary, there are onco-gynecological centers which report extensive PLN + PALN in EC patients, thereby increasing post-operative morbidity without an oncology safety increase (41). We focused on currently promising prognostic IHC markers ER, PR, L1CAM, and p53 mutation to determine whether these markers can help to refine the preoperative stratification of patients into high- and low-risk categories to assist the gynecological oncology surgeon selecting the adequate surgical extent.

A study published by van der Putten et al. revealed that L1CAM expression in curettage specimens is associated with features of aggressive endometrial cancer disease and poor survival of EC patients (42). van der Putten et al. (42) stated that L1CAM, ER, PR were associated with advanced stage, high-grade, non-endometrioid histology, lymphovascular space invasion (LVSI), and reduced disease-free survival (42). Trovik et al. (43) reported that combined ER/PR loss is a significant predictor of nodal affection and overall poor prognosis of patients. ER and PR are prospectively investigated in an ongoing study where the decision of whether to perform or not to perform lymphadenectomy is based on the pre-operational condition of hormone receptors (44). Prospective studies PIPENDO and PORTEC 4 are currently underway. The first study examines the use of molecular risk markers to identify high-risk patients requiring extensive surgery and/or adjuvant therapy (8). PORTEC 4 uses molecular risk factors for the stratification and indication of adjuvant radiotherapy (45).

In our study, ER, PR, L1CAM and p53 values from preoperative histology were related to definitive histology and grading. In endometroid carcinoma, there was a greater percentage of ER, PR receptors and no or ultimately low percentages of L1CAM mutations. The opposite ratio was seen in the occurrence of markers in non-endometroid ECs; p53 was mutated dominantly in endometrial grade 3 and non-endometroid carcinoma. The correlation of IHC markers with the extent of disease shows a decrease in ER and PR expression in higher stages of the disease. Furthermore, an increase in L1CAM expression can be observed when compared with early stages. Similarly, the p53 mutation was more common. Our results are in line with the published data in larger patient cohorts (43, 46). The correlation of markers with the presence of distant metastases could not be assessed as there were only two patients with distant metastases at the time of diagnosis in our study group.

To our best and honest knowledge, this is the first study to evaluate the added value of L1CAM, ER, PR and p53 markers in low- and high-risk EC preoperative diagnostics. This is the first study attempting to determine the cut-off of the individual markers for this classification.

We focused on the correlation of IHC markers with the determination of high-risk EC and the assessment of optimal cut-offs for continuous markers using ROC analyses. At the cut-off for ER <78% (*p* = 0.001), PR <88% (*p* = 0.008), and L1CAM ≥4% (*p* < 0.001), high-risk tumors were determined with a sensitivity of 28, 62, and 72%, respectively, and with respective specificity of 96, 72, and 86%. The PPV for ER, PR, and L1CAM were 87, 63, 83; the NPV for each marker were as follows: 59%, 65%, and 77%. The sensitivity of p53 mutated status (*p* < 0.001) for high-risk detection was low (34%), but the specificity was high (98%), which represents PPV 94% and NPV 61%, respectively.

A cut-off of 10% for positive L1CAM staining has been reported (6, 40, 47). van Gool et al. reported that when using a cut-off of 10% for positive staining, tumors in the study were classified as L1CAM-positive, with no significant association between L1CAM positivity and the rate of distant metastasis (*p* = 0.195). However, increasing the threshold for L1CAM positivity to 50% resulted in a reduction of the frequency of L1CAM-positive tumors and a significant association with the rate of distant metastasis (*p* = 0.018) (10). Estrogen receptors and PR are considered lost when expression is seen in <10% of the tumor cells. This cut-off used with breast cancer management in the prediction of hormone resistance was also evaluated in EC for prognosis prediction and published (42, 43, 48). In our cohort, we determined optimal cut-offs to distinguish low- and high-risk EC for ER <78% (*p* = 0.001), PR <88% (*p* = 0.008), and L1CAM ≥4% (*p* < 0.001). These cut-offs were established in a prospectively assessed cohort of consecutively included patients. In contrast to the cut-offs we defined, the published values are determined in retrospective cohorts of women with recurrence during follow-up, or with an adverse course of their disease with metastatic spread in parenchymatous organs and retroperitoneal lymph nodes. The incidence of L1CAM positivity and loss of ER, PR in these patients in retrospective cohorts may be significantly higher as it is an already pre-selected group of patients. The explanation may be based on the fact that patients who do not exceed the published cut-off values (10% for L1CAM, ER, and PR or 50% for L1CAM) but exceed the cut-offs set for the high-risk group in our study cannot be traced back in the retrospective studies, as they had not been radically treated with combined surgical and ± adjuvant therapy (radiotherapy, chemotherapy) and there was no recurrence during the long follow-up. Our results were confronted with 10 and 50% cut-offs for L1CAM; this setting led to good differentiation (high specificity) but at a very low sensitivity and good specificity.

To evaluate whether IHC markers would contribute to more accurate stratification of EC risk stratification, a new model was established. Parameters were obtained by imaging methods (myometrial invasion, cervical involvement, lymph node involvement), histology (endometrioid or mucinous vs. non-endometrioid), grade and IHC markers (ER, PR, L1CAM, p53). As deciding for risk stratification, the following parameters have been shown as crucial: L1CAM, PR, and myometrial invasion. The procedure of classification is shown in Figure 3. EC risk stratification's overall success was 78% for this model. Successful inclusion into a low-risk group was observed in 80% (56/70 patients), while for high-risk it was 76% (47/62 patients). We provided the comparison of current procedure represented by a model based on histotype, grading, and imaging methods with a new model consisting of imaging examination along with markers (IMG+IHC) (Table 6). IHC markers included in the current diagnostic would significantly improve sensitivity (48.4 vs. 75.8%, *p* < 0.001) associated with a slightly, statistically non-significant decrease in specificity to 80% (*p* = 0.238). Positive predictive value was similar for both methods, while negative predictive value (i.e., the probability of being true negative if the test is negative) was significantly improved (66 vs. 78.9%, *p* < 0.001), (Figure 4). Using our model, a significantly higher proportion of patients would be properly determined as high-risk.

When comparing the accuracy of the parameters used in the old model with the definite histology, we find discrepancies, especially in the grade and cervical invasion category (Table 1). At the present level of knowledge, no significant improvement in preoperative diagnostic accuracy can be expected by, for example, using imaging methods. Therefore, we are introducing a new model using molecular markers that are not dependent on imaging or other methods of clinical examination.

The strength of our study is that it is a cohort from a real clinical practice with prospective data collection and complete knowledge of preoperative and postoperative data. This is the first study to deal with the real implementation of new IHC markers in the pre-operational decision model. Our study design represents daily routine practice.

We acknowledge the study also has weaknesses. This is a relatively small cohort of EC patients, where all stages are not adequately represented; only two female patients in the FIGO IV stage were present. Considering the excellent correlation between preoperative and postoperative histology and grading, the weakness of the model is in its imaging method, which in the case of ultrasound is dependent on the expert skills of a particular sonographer or imaging specialist.

## Conclusion

We have demonstrated in our cohort that incorporating IHC markers into preoperative practice in endometrial cancer patients increases prognosis prediction accuracy and allows for the development of a new model for more accurate patient clinical management. Should we prefer a higher specificity model, then the most accurate classification is based on L1CAM values, myometrial invasion, and the condition of PR receptors. However, for wider implementation and the validation of proposed model, additional study is needed. Ideally, a prospective randomized trial would evaluate the role of IHC markers L1CAM, ER, PR, and p53 in a preoperative setting together with imaging method and histology/grade. To further improve the new model, it would be interesting to focus on general weaknesses in the accuracy of preoperative imaging methods and in the quality of preoperatively obtained samples with a full IHC examination on a routine daily basis. Incorporating IHC markers seems to be the best way to treat EC patients more accurately.

## Ethics Statement

The text of informed consent, information for patients and the study protocol was approved by Institutional Ethical Board of University Hospital Brno.

## Author Contributions

VW and MZ: idea of research project, overseeing, coordination of analyses; MB, PV, LM, and MF: collection and preparation of clinical data; JH, EJ, and MC: immunohistochemistry and interpretation of pathological data; PO: statistics; VW, MB, JH, PO, PV, and MZ: writing manuscript. All authors: final reading and approval of manuscript.

### Conflict of Interest Statement

The authors declare that the research was conducted in the absence of any commercial or financial relationships that could be construed as a potential conflict of interest.
